# Designing and Implementing Deliberative Processes for Health Technology Assessment: A Good Practices Report of a Joint HTAi/ISPOR Task Force

**DOI:** 10.1016/j.jval.2022.03.018

**Published:** 2022-06-01

**Authors:** Wija Oortwijn, Don Husereau, Julia Abelson, Edwine Barasa, Diana (Dana) Bayani, Vania Canuto Santos, Anthony Culyer, Karen Facey, David Grainger, Katharina Kieslich, Daniel Ollendorf, Andrés Pichon-Riviere, Lars Sandman, Valentina Strammiello, Yot Teerawattananon

**Affiliations:** Department for Health Evidence, Radboud Institute for Health Sciences, Radboud University Medical Centre, Nijmegen, The Netherlands; School of Epidemiology and Public Health, University of Ottawa, Ottawa, ON, Canada; Department of Health Research Methods, Evidence, and Impact (HEI), McMaster University, Hamilton, ON, Canada; Health Economics Research Unit (HERU), KEMRI-Wellcome Trust Research Programme, Nairobi, Kenya; Health Intervention and Policy Evaluation Research (HIPER), Saw Swee Hock School of Public Health, National University of Singapore, Singapore; Department of Management and Incorporation of Health Technology, Executive Secretariat of National Committee Health Technology Incorporation (CONITEC), Ministry of Health, Brasilia, Brazil; Centre for Health Economics, University of York, York, United Kingdom; Evidence Based Health Policy Consultant, Drymen, Scotland; Biointelect, Sydney, Australia; Department of Political Science, Centre for the Study of Contemporary Solidarity, University of Vienna, Vienna, Austria; Center for the Evaluation of Value and Risk in Health (CEVR), Tufts University Medical Centre, Boston, MA, USA; Institute for Clinical Effectiveness and Health Policy (IECS), University of Buenos Aires, Buenos Aires, Argentina; National Centre for Priorities in Health, Linköping University, Linköping, Sweden; European Patients’ Forum, Brussels, Belgium; Health Intervention and Technology Assessment Programme (HITAP), Ministry of Health, Bangkok, Thailand

**Keywords:** health technology assessment, deliberative processes, guidance, stakeholders, participation

## Abstract

**Objectives:**

Deliberative processes for health technology assessment (HTA) are intended to facilitate participatory decision making, using discussion and open dialogue between stakeholders. Increasing attention is being given to deliberative processes, but guidance is lacking for those who wish to design or use them. Health Technology Assessment International (HTAi) and ISPOR—The Professional Society for Health Economics and Outcomes Research initiated a joint Task Force to address this gap.

**Methods:**

The joint Task Force consisted of 15 members with different backgrounds, perspectives, and expertise relevant to the field. It developed guidance and a checklist for deliberative processes for HTA. The guidance builds upon the few, existing initiatives in the field, as well as input from the HTA community following an established consultation plan. In addition, the guidance was subject to 2 rounds of peer review.

**Results:**

A deliberative process for HTA consists of procedures, activities, and events that support the informed and critical examination of an issue and the weighing of arguments and evidence to guide a subsequent decision. Guidance and an accompanying checklist are provided for (i) developing the governance and structure of an HTA program and (ii) informing how the various stages of an HTA process might be managed using deliberation.

**Conclusions:**

The guidance and the checklist contain a series of questions, grouped by 6 phases of a model deliberative process. They are offered as practical tools for those wishing to establish or improve deliberative processes for HTA that are fit for local contexts. The tools can also be used for independent scrutiny of deliberative processes.

Health technology assessment (HTA) bodies around the globe continue to evolve their processes for better-informed decision making.^1^ One recognized approach to improving the quality of recommendations and decision making is through the use of deliberation. Deliberation enables exchange between those deliberating through participatory processes that emphasize reasoning for the purposes of deepening the understanding of, or providing critical insight into, differences between participants, including how these might be resolved. Deliberative processes consist of procedures, activities, and events that support deliberation in HTA. They have been promoted in healthcare priority setting since the 2000s (eg,^2–4^).

Whereas most HTA bodies elicit stakeholder views and/or consult experts on the design or conduct of the HTA, the focus in deliberation lies on stakeholder participation using discussion and open dialogue. Well-conducted deliberative processes can make explicit the assumptions, arguments, and values that are entailed when assessing health technologies (eg, importance of equity of access). They can also reveal additional information about facts (eg, organizational challenges), which may have practical significance if decisions are to be successfully implemented.

A recent set of core values and supporting actions for deliberation in HTA has been proposed.^5^ However, the core values and associated actions were developed with drug reimbursement recommendations specifically in mind, so their applicability to other stages of HTA and health technologies is unclear. Likewise, specific guidance for legitimizing health benefits package design has been developed.^6^

Building on these specific initiatives, a joint Task Force, coled by Health Technology Assessment International (HTAi) and ISPOR—The Professional Society for Health Economics and Outcomes Research, was initiated, providing comprehensive guidance and an accompanying checklist for (i) developing the governance and structure of an HTA program (ie, deliberation about processes) and (ii) managing the various stages of an HTA process (ie, deliberation within processes, based on,^7^ see Box 1).

The process of creating guidance and accompanying checklist was based on ISPOR’s “Good Practices Reports.”^8^ A proposal developed by the appointed cochairs (W.O. and D.H.) was reviewed and approved by both the HTAi and ISPOR Board of Directors in May 2020. Task Force members were invited by the cochairs based on expertise, geography, and work environments. The guidance was developed through monthly Task Force member interaction until December 2021, and input was sought on practical examples via HTAi’s interest groups. Intermediary results were presented and discussed with HTA experts and representatives from HTA bodies during organized HTAi and ISPOR panel sessions in June and July 2021. Finally, peer review of the draft final report was conducted through 2 formal rounds of written review (see the Acknowledgments section).

Where possible, we have provided illustrative examples from existing HTA practice or the literature. However, we recognize that documented deliberative processes for HTA are still evolving and are rarely fully observed in actual operation. We hope that our report will persuade those interested in the value of deliberation and make it easier to design and implement suitable processes that are fit for local contexts.

## Target Audience

The target audience for this guidance is the executive and legislative actors responsible for establishing and managing HTA processes, particularly HTA bodies. We recognize that HTA administrators may have different degrees of autonomy and independence, and that their accountability is based on existing legislation or regulation, as well as on societal expectations and norms. We are, therefore, providing guidance broad enough to be applied to any stage of a process related to HTA, and flexible enough to allow them the opportunity to create and defend their individual approaches.

A secondary audience for the guidance is interested stakeholders: people who are keen to understand both what deliberative processes are and how they might better participate in those processes if the opportunities are available. It can also be used by researchers: people researching the quality or completeness of a deliberative process or seeking to compare these processes across HTA bodies.

## Definitions and Scope

As there is no widely recognized definition of a deliberative process for HTA, the first goal of the Task Force was to create a consensus definition. Existing definitions of “deliberation,” developed for health policy or other related activities (eg,^9,10^) did not appear satisfactory for our purposes. They were too focused on a particular application of the concept for one form of HTA, for example, pharmaceuticals. They also excluded some items suitable for deliberation, such as agendas for meetings, which may be appropriate in specific contexts but ought not to be ruled out by definition. They also missed the importance of exploring the political, economic, and social context in which HTA is to be applied. The Task Force, therefore, developed the following definitions (Box 2).

Those designing and supporting deliberative processes for HTA should not underestimate the importance of selecting and clearly stating the desired goals and outcomes of deliberation, as these will determine all other design choices.^11^ Deliberation can, for example, be used to provide an opinion (eg, advice or recommendation), but can also be used to understand (diverging) views regarding a specific issue. Deliberation can create opportunities for exposing possibly conflicting values and perspectives and reaching resolutions that will be useful to ultimate decision makers (including resolutions that are not unanimous).

These values and perspectives are typically provided by a variety of stakeholders (patient[s], public/citizens, providers of care, payers, producers and innovators of health technology, principal in-vestigators in research, and policy makers) and experts (eg, experts in medicine, law, ethics, economics, epidemiology, bioengineering, and patient-based evidence). Stakeholders and experts can have financial, professional, or reputational interests in the outcomes of deliberative processes, which points to the need for their roles to be clearly defined. An HTA body must then decide how these perspectives will be represented, how participant views are exchanged, how participants are identified and selected, how partiality and bias are to be minimized, and whether and how the identities of participants are to be publicly disclosed.

Good deliberation includes the consideration and examination of general factors, such as the identities of participants, their roles, key decision criteria and possible conflicts between them, the scope of costs and benefits and their measures, the measurement and significance to be attached to factors, such as equity and public acceptability, and more specific factors related to a specific health technology, such as the evidence for its best use, its precise and accurate interpretation, and—when this is the goal—the dissemination and implementation of the output(s) of deliberation. These and other design and implementation considerations are part of the checklist for deliberative processes for HTA (Table 1) and elaborated upon after introducing the checklist and how to use it.

## The Deliberative Processes for HTA Checklist and How to Use It

The checklist (Table 1) is intended to be used prior to designing an individual deliberative process or when reviewing an existing one. It is intended to be completed for each new process, whether for developing the governance and structure of an HTA program or managing one or more different HTA-related stages.

The checklist contains a series of questions, grouped by 6 phases of a model deliberative process. Users of the checklist should simply check all answers that apply to each question posed. The answers represent a minimum set of considerations and design features that are required in order to qualify a process as deliberative. The checklist is not intended as a scoring tool, but allows users to identify items that are not checked, and more readily scrutinize whether improvements can be made.

Some questions relate to the key features of a deliberative process that should be stated in its terms of reference. These questions are labeled with an asterisk “*” in the checklist. Asterisked items may also be considered a minimum set of features for any process to be considered and can be used for comparisons across processes.

## Guidance for Designing and Implementing a Deliberative Process

### Phase I Determining the Need for a Deliberative Process

The need for a deliberative process depends on its goal(s), the desired outcome(s), and scope.

#### Why Deliberate?

Deliberation allows participants to exchange their views and perspectives. Participants may learn about other views and perspectives as a result of deliberation, leading to predeliberative opinions being updated or revised. Participation through deliberation also gives some degree of influence and ownership of the final output to those involved. Deliberation can lead to an expansion of what counts as evidence in the process. Well-designed and well-executed deliberative processes also have the potential to provide stakeholders with reasons to perceive the HTA process as (un)fair, based on its procedural characteristics (eg, inclusivity of all relevant stakeholders), even if they disagree with the outcomes of the process.^2,10,13,14^ It may yield outputs that are more inclusive, better framed, more balanced, and more feasible. This will increase trust in institutions and their decisions.^15^

#### What Are the Desired Outcomes of the Deliberative Process?

HTA bodies might consider several desired outcomes related to the goals of deliberation. These include (i) the sharing and potential shifting of different viewpoints among participants, (ii) reaching common ground that underpins a collective decision; (iii) an opportunity to provide reasons for and against outputs, and a possible revision of them following deliberation; and (iv) strengthening mutual respect and understanding among participants. Although agreement can also lead to a collective opinion (eg, advice, recommendations, or decisions), this is not always the required output of a deliberative process.^2,10,14^

#### What Is the Scope of Deliberation?

Deliberation can inform a range of processes within an HTA body (Box 1), from formal discussions of an HTA organizational structure (deliberation about processes) to topics for assessment and the revisiting of an earlier decision on a specific health technology in the light of new evidence (deliberations within processes).

##### Deliberation about the HTA Process

Deliberation about the HTA process will be shaped by historical, sociocultural, political, economic, and institutional context for healthcare decisions, and these will (need to) be reflected in the autonomy, mandate, capacity, and governance of an HTA body.^16^ Therefore, whether (and at what stage) to deliberate will likely be driven by judgments about its (potential) use or effectiveness given institutional attitudes toward independence, transparency, and inclusiveness.^17^ Decisions like these may be informed by specific actors, such as regional administrators or ministries of health. In Canada, for example, Abelson et al^18^ have developed a public and patient involvement framework for an HTA body’s process that addresses decision making occurring at a provincial level. The depth and complexity of actions (to be) undertaken by an HTA body may also dictate what degree of deliberation is required.^19^

##### Deliberation within the HTA Process

Deliberation is needed or more desirable when there are anticipated and potentially controversial differences in what facts are relevant to the deliberation, how empirical information ought to be interpreted and valued, as well as what values are relevant to the decision and what these imply regarding the collection of information to inform deliberation; this may apply to the HTA process, as described above, as well as at several HTA stages.^15,20^ Examples of deliberation at different HTA stages are described in Box 3.

The benefits of deliberation must be balanced against the possible costs, the capacity to manage it, and timelines for decision making. The benefits of achieving a certain degree of rigor in deliberation may be costly, and it may be unnecessary or make a particular model infeasible. In addition, contextual factors (eg, who is accountable for the process overall and to whom, available HTA content knowledge, and perceptions of conflict) may facilitate or hinder the feasibility of implementing deliberative processes and limit their contribution to legitimate decision making. For these and related reasons, what might be the most important aspects of a deliberative process to invest in can be determined by using the checklist (Table 1).

### Phase II Preparing for a Deliberative Process

Contextual factors, guiding principles, as well as what should be documented and communicated to the public are important considerations when preparing for a deliberative process.

#### What Contextual Factors Are Relevant to the Deliberative Process?

Internal and external contextual factors influence the type and scope of deliberative processes that can be implemented in any setting.^26^ *Internal factors* include governance, leadership and organizational culture, financial constraints, availability of local information, availability of participants, the knowledge and skills of applying HTA methods, and the capacity or financial resources to conduct HTA and communicate HTA decisions.^27^ *External factors* include the extent to which countries have established mechanisms to incorporate evidence into decision making, the perceived role of HTA in decision making, and the mandate given by legal or policy authority.^27^

Another important external contextual factor is influence by interest groups, including politicians, ministers of finance, industry, patients, and care providers. This has led to a wider range of stakeholders being involved in HTA processes and pressure to consider a broader range of criteria in decision making.^28^ Poorly implemented deliberative processes that do not manage these power dynamics can lead to distractions that dilute fruitful discussion.^29^ This creates ethical challenges for patients^30^ as well as risks of delay or poor decision making (see Box 4,^31^).

There are also contextual factors specific to a decision about use of a health technology that is established during the deliberation about the technology itself (see Box 5).

Having an HTA infrastructure that supports transparency, participation of relevant stakeholders, and accountability of decision makers will be an important minimum requirement for enabling deliberation in any setting.^27^ We cannot assume that the implementation of deliberative processes automatically improves decision making across all jurisdictions equally. Variation in internal and external factors will shape and constrain implementation. Identifying factors that may hamper the effectiveness of a deliberative process enables planning to mitigate or overcome these barriers.

Ways of mitigating these barriers include policy statements on the willingness to use HTA in policy or practice, and development of transparent procedures for deliberations even if the outputs need to remain confidential, such as the communication of who is involved, their conflict of interest declarations, and how the process is undertaken.

There is an opportunity among countries with less-developed deliberative processes to start building them with features that take account of decision-making needs and environmental and societal constraints. Most low- and middle-income countries are in the early stages of developing HTA systems and can potentially incorporate learnings from the experiences of others.^6^ In contrast, most high-income countries have already established institutions and processes that may be more difficult to change.^33,34^ This means that these countries will require approaches to evaluate their current deliberative processes and to identify where improvements might be made.

#### What Are the Guiding Principles?

Principles are intended to guide the design and conduct of deliberative processes. They can be substantive or procedural.^35^ Efficiency in HTA, for example, is an important principle where resources for HTA are lacking. Walton et al^36^ reported, for example, that the National Institute for Health and Care Excellence (NICE) judged the resources dedicated to discussing individual topics to be not efficient and unsustainable.

To be useful to decision making, principles should be consistent with the overarching goals of the HTA body or healthcare system that the deliberative processes are intended to support (eg, equal access to care). Such goals are often multidimensional, complex, and value-laden. Values such as transparency, impartiality, and inclusivity were recently identified as important procedural principles related to deliberations about drug coverage decisions.^5^ Other values may include timeliness, consistency, and verifiability (eg, making recommendations that are timely and understandable).14 Values can be differently applied to the various stages of an HTA process and may conflict. An example of the potential conflict between values of transparency and impartiality in developing drug funding recommendations is shown in Box 6.

The selection and application of principles and their related values may be affected by local contexts as different decision makers may have different goals, work in different sectors, and hold different attitudes toward (the need for) deliberation.

In some countries, technology producers implicitly determine the topics for assessment by submitting reimbursement decision dossiers to HTA bodies throughout the year, whereas in other topics are nominated and selected with the involvement of patients, payers, ministry of health officials, healthcare managers, and clinical experts.^37^

#### What Should Be Documented and Communicated to the Public?

The need for a deliberative process, the guiding principles, and key elements for conducting and supporting a deliberative process as reflected in terms of reference, its outputs, and how they are used, as well as the monitoring and evaluation thereof (see sections below), need to be documented and communicated to the public. This documentation will also aid in sharing and studying what design features work best, and assist in promoting standards for HTA bodies in years to come.

### Phase III Conducting a Deliberative Process

When conducting a deliberative process, it is important to consider several design features, including the selection and identities of participants (including the chair[s] or facilitator[s]), openness, type, length, and the rules of deliberation.

#### Who Deliberates?

Participants in a deliberative process may include stakeholders, experts, or both. Some participants may be asked to play more than one role. For example, an industry representative is both a stakeholder affected by a decision, but may also have expertise in trial design and interpretation of evidence within a therapeutic context. The mix of participants and approaches to recruiting them should be carefully considered and will depend heavily on the tasks to be completed. Participation could take the form of membership of a standing committee where patient representatives have the task to represent their personal experiences or represent the wider experience of a group of patients. There might be *ad hoc* deliberative processes on selected topics in which a wider range of stakeholders (eg, industry groups, healthcare payers, clinical experts, and patients) participate.

Other models of representative deliberation exist (eg, consensus conference, citizen forum, and platform for representative deliberative democracy [G1000]^38^), and may be more feasible or better for meeting the goals of the HTA process. In The Netherlands, a deliberative citizen forum has been used by the Zorginstituut Nederland (ZIN; the national HTA body) to obtain insight into the criteria-informed citizens would propose for public reimbursement.^39^ Even within the same political context, participants involved in deliberation may vary according to what is being deliberated, and the goals and intended outcomes of deliberation (see Box 7).

The number of participants should be dictated by the goals and principles of a deliberative process. Although 10 to 15 participants have been suggested as an effective number for multidisciplinary advisory committees,^40^ others have stated, “there is no magic number that constitutes the ideal-sized group.” Trade-offs between quality and speed must be made.^41^ In the case of controversial HTA questions (eg, when major ethical issues are raised by the health technology that is being investigated), a larger group—including all relevant perspectives—that takes more time to deliberate (and may have higher quality deliberation) may be more desirable for engendering trust and legitimacy.

In practice, HTA bodies operate with standing deliberative committees consisting of 9 (ZIN in The Netherlands) to 29 members (Haute Autorité de Santé [HAS] in France). HTA bodies should balance the need for representativeness with administrative burden and group effectiveness. The form and composition of the group and (if applicable) who has voting rights will also vary according to its mandate or legislation. Publicly stating who is participating, their interests and how conflicts will be reported will enhance the perceived legitimacy of the process. A common approach to support effective participation in deliberative processes of HTA bodies is asking all participants to declare their interest(s) prior to each meeting.^42^

#### What Membership Arrangements Enable Effective Deliberation?

Although an *ad hoc* group may be suitable for the purposes of deliberating about HTA processes, deliberation within processes typically involves one (eg, in Brazil and The Netherlands) or more (eg, in Australia, France, and Thailand) standing committees. A hybrid form can also be used. For example, in Germany, patients and representatives appointed by the Conference of Health Ministers of the German States have discussion and petition rights on all agenda items of the Federal Joint Committee (standing committee) meetings. In addition, several associations have representation at meetings with participation rights on specific topics.

#### How Will Participants Be Selected?

Identifying and selecting participants (stakeholders and experts) can be accomplished through an open process and interviewing (as done in The Netherlands) or by targeting groups with informative perspectives (as done in Brazil). HTA bodies may use advertising or invitations to accomplish either approach.

#### How Are Perspectives to Be Represented?

Participants may represent the views of others or only express their individual views.^43^ Depending on the topic(s) under discussion and the stage at which it is being deliberated, an individual stakeholder or expert could also be considered an active participant in deliberation, or alternatively, simply as a provider of information to support deliberation by others (ie, through consultation). HTA bodies need to decide how to include harder-to-obtain or often underrepresented perspectives (eg, from minority groups) and how these views inform the output(s) of deliberation (ie, whose votes count and with what weight regarding the recommendation or decision).

Participation of patients as well as public/citizens in the HTA process is also increasingly being considered. Despite the differing perspectives offered by individuals in these roles,^44^ there has been a tendency to consider patient and public/citizen roles together.^45^ There is also considerable uncertainty regarding how best to capture both perspectives, and efforts continue to establish best practices in this area.^13,18,45^ Challenges with patient involvement have been identified, and include ethical issues related to coercion and selection bias when only the most strongly motivated patient representatives participate. For example, a recent analysis of patient representation at the Canadian Agency for Drugs and Technologies in Health (CADTH) highlighted concerns about a lack of attention to preventing some patient voices from “dominating the process” over others.^46^

Public involvement may be an even more challenging perspective to represent. Some HTA bodies have used separate citizens’ juries or panels to inform expert discussion,^47^ whereas others have attempted to have the public perspective represented on advisory committees. For public involvement, factors identified as being critical ingredients to effective deliberation include (i) credibility, that is, an ability to contribute knowledge that is considered valid and relevant and that will result in mutual learning and generation of new solutions; (ii) legitimacy, that is, an ability to speak on behalf of others affected by healthcare decisions; and (iii) power, that is, an ability to influence healthcare choices.^48^ An exploration of public expectations in Australia revealed the public wanted HTA processes to “include a diversity of individuals, be independent and transparent, involve individuals early in the process, and ensure that public input is meaningful and useful to the process.”^44^

#### How Will Participants’ Identities Be Disclosed?

HTA bodies must decide if and how participants’ identities will be disclosed. Disclosing who is involved, their interests, and how conflicts will be decided and settled will enhance the perceived legitimacy of the process. HTA bodies should also consider that full transparency on these issues could lead to privacy concerns for some participants, including undesired media attention or pressure from interest groups.^49^

#### How Open Should the Deliberation Be?

HTA bodies must decide how open the record of deliberation should be. Deliberative processes can be open to the public (eg, ZIN in The Netherlands and the Institute for Clinical and Economic Review in the United States), closed (eg, HAS in France; prioritization working group organized by the Health Intervention and Technology Assessment Programme [HITAP] in Thailand), or a mix (eg, the NICE in England). Although an open process will result in more transparency, HTA bodies should also consider that full transparency could lead to strategic behavior of some participants.

#### What Type and Length of Deliberation Is Needed?

The mode of deliberative processes may be virtual (eg, in Brazil, Scotland, England, and The Netherlands), face-to-face, or both. Participants may exchange views at a single meeting, at several meetings, at the same time, or at different times. The value of face-to-face deliberation may improve communication among participants, although better communication must be balanced against the potential for undue influence by dominant participants to minimize poor decision making by groups.^50^ These can include nominal group techniques, consensus-building approaches, and expert elicitation techniques. However, these approaches are seldom used by HTA bodies.^51^ HTA bodies should decide what level of rigor for deliberation is required and what is feasible given the available resources.

#### What Are the Rules of Deliberation?

Establishing rules of deliberation requires consideration of the goals of the process, who is participating, and how they will be engaged. Rules of deliberation can encompass both rules regarding how participants interact and share views, over what time frame, as well as rules for what specific information or topics can be discussed. The rules of deliberation are far more influential on affecting the outputs of deliberation than the participants involved or the information considered.^52^ The rules of deliberation may also be of particular importance when there are multiple deliberative forums and a perceived need for consistency in approaches.

For those deliberative processes intended to provide an opinion (eg, advice or recommendation), rules are important regarding what procedure will be used to lead to the conclusion of deliberation. For example, for drug coverage decision making, consensus building (eg, in The Netherlands), majority voting (eg, in France and Scotland), or both (eg, in Australia, Brazil, and England) are used by HTA bodies. Deliberative processes intended to result in an opinion may need to consider how disagreement is managed, which could include voting rules. In contrast, processes related to understanding (diverging) views, establishing expectations, creating narratives, and empowering patients and communities will more strongly emphasize participatory dialogue approaches.

### Phase IV Supporting a Deliberative Process

Supports for deliberation include (i) organizing interaction to create participant perceptions of meaningful interaction,^53^ (ii) effective communication of information to facilitate deliberation,^54^ and (iii) providing comprehensive terms of reference. These supports not only serve to make deliberation effective, but are also intended to empower participants.

#### How Will the Exchange of Viewpoints Be Facilitated during the Deliberative Process?

Power differences between participants, including gender, wealth, ethnicity, organizational seniority, and education, have been highlighted as challenges to effective deliberation.^53,55^ Addressing power differences requires, in part, an adequate exchange of views between participants. This is often done through a central agent (eg, chair[s] or facilitator[s]) whose role is to identify preferences and underlying beliefs of all participants, confront others with these, and work toward a mutual understanding of the issue(s). Effective deliberation also requires participants to understand the process.

A significant concern is that participants may undergo social pressure to conform to the wishes of the group and leave the process unsatisfied.^52^ They may also be susceptible to cognitive or confirmation biases, that may be further subject to social influences.^56^ As decisions may be swayed by allowing one member of a group to dominate, the importance of rigorous and balanced facilitation or approach to exchange cannot be underestimated.^57^

#### What Information Will Be Made Available?

HTA bodies need to consider the amount and type of information made available to participants, when it will be made available, as well as how it is gathered, interpreted, and communicated. The information provided will depend on the HTA stage.

For example, for topic selection and prioritization at the NICE (England), all participants receive similar written information, and they are backed up by clear process documents or standard operating procedures (in addition to Terms of Reference). When contextualizing HTA, some countries provide evidence synthesis using structured guidance (eg, the Grading of Recommendations Assessment, Development, and Evaluation (short GRADE), as used in Germany; https://www.iqwig.de/methoden/general-methods_version-6-0.pdf), whereas others provide a draft HTA report (eg, Scotland and England), or provide a draft HTA report with the reactions and perceptions of stakeholders on the draft report clearly identified (eg, in The Netherlands).

To ensure an appropriate amount of scrutiny, consideration should be given to presenting a complete package of information to all participants in a deliberation, rather than presenting redacted or interpreted summaries, which are subject to error and may reduce perceptions of legitimacy. This information could include original clinical studies in addition to a synthesis report (as done at the CADTH in Canada), an original economic model in addition to a summary report (as done at the NICE in England), or other relevant information (eg, patient-based evidence gathered through interviews, clinical reviews, and assessment checklist results).

If specific criteria are used to guide a decision or recommendation, an HTA body may additionally provide standardized summaries or lists that address these. Checklists, similar to standards used in published research^58^ but tailored to a specific jurisdictional context, may also be useful. To further reduce errors when interpreting or synthesizing information for participants, the use of standard analytic judgments (eg, consensus guidance for analysis, such as in Australia) could be considered. Participants will also benefit from an overview of what information is missing as well as the limitations of interpreting available information.

#### What Information Will Be Considered by Participants and How Will Information Be Reviewed and Revised?

Just as allowing dominant viewpoints can be a threat to meaningful participation, so can the introduction of information that has not been equally considered by all participants. This can be remedied by creating enforcing rules regarding what information is allowable (ie, not allowing participants to introduce new or modified, and potentially out-of-scope, information during deliberation).

#### How Will Information Be Presented?

Meaningful participation requires a clear and common understanding of available information and its interpretation.^55^ Effective participant understanding of presented information, including scientific evidence, is facilitated by the use of appropriate media (written, verbal, or both), reporting structure(s), clarity and level of language used, and ensuring a mutual understanding of how the information is identified and synthesized.

In presenting complex, technical information, HTA bodies will need to consider levels of health literacy among participants and introduce appropriate supports (eg, training or briefing sessions) to ensure exchange that is not hindered by avoidable misunderstanding. This could also include standardized approaches to producing lay summaries and allowing participants sufficient time to clarify misunderstandings prior to deliberation. In England, NICE’s Public Involvement Programme provides direct support and training to patient and carer consultee organizations, their representatives, and individual lay persons, patients, or carers. This enables them to contribute to the discussions during deliberative meetings (https://www.nice.org.uk/about/nice-communities/nice-and-the-public/public-involvement/public-involvement-programme).

#### What Specific Supports for Participants Are Available?

HTA bodies will need to consider if and what remuneration they might provide to participants in deliberation as this may lead to more willingness to participate and better engagement. They can follow governmental policies on financial compensation to serve at committees of public agencies, as done in Canada, Poland, The Netherlands, and England.^42^ Better engagement can also be facilitated through training and education to orient participants to the deliberative process as done by the National Committee Health Technology Incorporation in Brazil and the HITAP in Thailand.

Tools and checklists may aid effective deliberation. A checklist for HTA bodies to design their efforts for meaningful stakeholder participation has been developed.^12^ Wale et al^59^ also provide good practices more narrowly focused on patients and public perspectives and expertise in HTA deliberative processes. Examples include the participation of patient experts to answer questions during the contextualization process of the Scottish Medicines Consortium in Scotland. HTAi has also developed relevant tools and guidance to support patient organizations in completing a patient group submission template (https://htai.org/interest-groups/pcig/). In Australia, the International Association for Public Participation^60^ and the Patient Voice Initiative have similarly developed standards and online tools for patient groups and communities when making submissions to the Pharmaceutical Benefits Advisory Committee’s (PBAC; https://www.patientvoiceinitiative.org/resource-library/). In the United States, the National Health Council has a range of resources to support patient input to “value assessment” including basic HTA training (https://nationalhealthcouncil.org/education/value-classroom/).

#### Are Comprehensive Terms of Reference in Place?

To enhance public trust and be transparent about the key aspects of (conducting) a deliberative process, comprehensive terms of reference should be created and be publicly available. A term of reference defines the purpose, scope, outcomes, and structures of a deliberative process. We recommend that questions labeled with an asterisk “*” in the checklist (Table 1) are addressed in such a document and reviewed periodically (eg, every 3–5 years).

### Phase V Development and Communication of the Outputs of Deliberation

Reporting and communicating the process of deliberation, its output, as well as mechanisms for reconsideration of the outputs are important design features to consider.

#### How Will the Process and Output(s) of a Deliberation Be Reported and Communicated?

Documenting the process and output(s) of deliberation is the responsibility of the organization that is establishing or managing HTA processes and can be facilitated through video (livestreaming or published recording) and by taking meeting minutes. Meetings can be reported back to all relevant stakeholders in entirety or as a summary, for example, by using a standardized format that includes an explicit description of the issue, the information used, and the views and argumentation that have been put forward. A rapid communication of the output(s) (eg, recommendation) to an organization submitting a health technology dossier is an important part of processes where appeals or resubmissions are an option (see below). On the other hand, rapid, abbreviated reporting can lead to information loss of specific considerations.^48^ Providing a complete record can facilitate a better understanding of situation-specific considerations that may arise within deliberation for all stakeholders not involved in the deliberation.

#### How Will the Outcomes of Deliberation Be Reconsidered?

Possible mechanisms for reconsideration include appeal, revision, and additional deliberation. The scope and process related to any of these mechanisms may vary depending on legislative or regulatory frameworks. Some HTA bodies, such as the Health Insurance Review and Assessment Service [HIRA] in Korea,^61^ the NICE in England, and the PBAC in Australia have established mechanisms or guidance for lodging an appeal (see Box 8 for the process at the NICE).

Appeal refers to a mechanism that gives stakeholders the possibility to apply for a revision of a recommendation, involving arguments offered by the individual or group making an appeal, and leading to a reasoned response.^62^ In designing appeal mechanisms, it is important to be explicit about what constitutes grounds for appeal and/or revision, the characteristics of the body in charge of the appeal (eg, whether it is independent and the members of the appeal committee),5 who can or cannot appeal, how the appeal will be considered and decided, and the timelines involved.

For deliberative processes that do not result in a decision, a review and revision of viewpoints, including additional deliberation, may be warranted, particularly if new information from external experts and stakeholders is developed and presented.

#### How Will the Final Output(s) of Deliberation Be Communicated?

A communication strategy should include what, when, and how the output(s) will be communicated and to whom, and be publicly available. Such a strategy could be aimed at communicating the output(s) of deliberation and how it was arrived at. Some suggestions for reporting are provided in Box 9.

The responsible organization should make efforts to ensure that output(s) of deliberation are well communicated and target all relevant stakeholders, using a variety of channels and approaches. These may include the use of official documents (eg, an official journal), Web sites of relevant organizations, policy briefs, newsletters, and news items on social media to address the broader public.

Consideration may also need to be given to the kind of information and different levels of detail for different audiences. For example, a health technology developer may expect a greater level of detail related to the assessment of evidence and uncertainty, in order to plan for any resubmission or appeal, whereas a patient may be more interested in the implications of the decision for access and possible next steps in that direction.

### Phase VI Monitoring and Evaluation

Monitoring and evaluation are necessary to determine if a deliberative process is achieving its intended aims, to identify aspects that are being done well, and ways in which the process might be improved.

#### Is the Desired Change(s) from Implementing a Deliberative Process Established and Does It Align with the Health System Values or Principles Established and/or with Those of the HTA Body?

HTA bodies should start with a comprehensive description of how a desired change is expected to happen from a deliberative process in their context, including the resources (inputs) needed along with the rationale for such change. HTA bodies may assess whether the health system values and guiding principles of deliberation are upheld. The outcomes of a deliberative process could be assessed to evaluate the extent to which the desired change has been established.

#### How Will the Desired Change(s) from Implementing a Deliberative Process Be Measured?

Questionnaires, interviews,^63^ document reviews, or live meetings held with participants are some mechanisms to evaluate deliberative processes. The HTA body can ask for feedback on the process of how the evidence report has been developed, and how experts and stakeholders perceived the process in terms of fairness, transparency, and timeliness, as well as the level and impact of participation.^64^

HTA bodies may also consider assessing the outcomes of deliberation that are tied to the broader aims of HTA, such as distributional justice in health care or overall improved health.^5^ It is important to note that certain outcomes may not be achieved immediately, not because the processes are ineffective but rather due to a time lag for measuring the impact after implementation. It must also be recognized that both process (inputs and activities) and outcome measures are interrelated, with the inputs and activities mainly informing short-term success, whereas the outcomes reflect longer-term impact.

#### How Will the Desired Change(s) from Implementing a Deliberative Process Be Assessed?

Monitoring and evaluation require considerations of who does the assessment, how it can be done, and what tools can be used. In addition to involving participants in deliberative processes, there are advantages to seeking an external party, such as an independent evaluator, who may provide an unbiased perspective to the evaluation. Who to involve largely depends on how monitoring and evaluation are done, whether monitoring is done on an *ad hoc* basis, or more routinely and embedded within a broader framework for evaluating other components of HTA. The monitoring and evaluation approach can be integrated into an existing monitoring and evaluation framework of the HTA body. The HTA body may consider developing or adapting established tools, such as checklists or questionnaires for eliciting feedback. Alternatively, using qualitative methods, such as focus groups and in-depth interviews with key stakeholders either directly or indirectly involved in deliberative processes may be considered for *ad hoc* assessments.

#### What Indicators Will Be Used to Monitor and Evaluate the Deliberative Process?

In Table 2, we suggest indicators, stakeholders to be involved, and methods for monitoring and evaluation of deliberative processes. These are by no means comprehensive. It is recommended that those leading the monitoring and evaluation adapt them and develop context-specific measures for each, which may be qualitative or quantitative. The process by which these are defined may also be done through deliberation.

### Improving Deliberative Processes for HTA

The guidance and accompanying checklist are offered as practical tools for those wishing to establish or improve deliberative processes for HTA that are fit for local contexts. They can also be used for independent scrutiny of deliberative processes.

As deliberative processes for HTA are rarely seen in actual operation, we encourage those that (intend to) use the checklist and guidance or have recently implemented a deliberative process to share their experiences with the corresponding author of this report. We will use this information to further optimize the guidance and accompanying checklist and to inform future activities of the Task Force.

## Figures and Tables

**Table 1:** Deliberative Processes for Health Technology Assessment (HTA) Checklist

Phase	Question	Details	Notes
Determining the need for a deliberative process	Why deliberate?* What are desired outcomes of the deliberative process?* What is the scope of deliberation?*	Goals of deliberation may include: To generate additional information □ To probe and explore the values under-pinning posi-tions taken □ To reduce influence of selfinterest □ To optimize HTA processes □ To comply with legal requirements □ To improve the acceptance of decisions □ To improve the perceived legitimacy of the HTA process □ To enhance public trust □ Other, please specify □ Sharing and potential shifting of participant reasoning or viewpoints □ Reaching common ground □ Revealing divisions or dissent among participants □ Better mutual respect and understanding among participants □ About HTA processes (designing HTA/decision-making processes) □ Within HTA processes ○ Identification of topics (eg, horizon scanning) □ ○ Prioritization of relevant topics for HTA □ ○ Providing scientific advice □ ○ Scoping, assessment and synthesis of relevant information □ ○ Contextualization of HTA □ ○ Development and communication of the output(s) monitoring and evaluation □	These are the ultimate “goals” of implementing a deliberative process about or within HTA processes or stages. The desired outcomes should relate to the goals. Deliberation must be fit for purpose. Any new deliberative process should have a well-defined scope.
Preparing for a deliberative process	What contextual factors are relevant to the deliberative process? What are the guiding principles? * What should be documented and communicated to the public?	Internal factors ○ Governance □ ○ Allocated budget □ ○ Availability of local information □ ○ Availability of stakeholders □ ○ Awareness of analytic methods □ ○ Capacity or financial resources to conduct and communicate deliberation □ ○ Other, please specify □ External factors ○ Mandate given to organization □ ○ Constitutional rights □ ○ Existence of mechanisms to use evidence in decision making □ ○ Perceived role of HTA in decision making □ ○ Societal norms for public and private discourse □ ○ Other, please specify □ Guiding principles are in place and oriented to the overarching goals of HTA body or healthcare system □ Or, consider guiding principles driven by values such as: ○ Transparency □ ○ Impartiality □ ○ Inclusivity □ ○ Timeliness □ ○ Consistency □ ○ Verifiability □ ○ Efficiency □ Other, please specify □ A complete documentation might include: Comprehensive terms of reference (for the items marked* in this checklist) □ Documentation of how the outputs of deliberation were developed and communicated □ Documentation of the monitoring and evaluation process □ This completed checklist □	Recognizing contextual factors that can hamper deliberative processes enables planning to limit or overcome these risks. This can also be used to clearly state (both externally and internally) why aspects of a deliberative process are not (yet) able to be implemented. Principles are intended to guide actions or decisions related to the design and conduct of deliberative processes. Values underlying principles, such as those listed, may conflict and require trading off, possibly through deliberation. The deliberative process, and how it was determined, in turn, can also then be communicated to others affected by those decisions, to further aid in supporting its legitimacy.
Conducting a deliberative process	Who deliberates?* What membership arrangements enable effective deliberation?* How will participants be selected?* How are perspectives to be represented?* How will participants’ identities be disclosed?* How open should the deliberation be?* What is the type of deliberation needed?* What is the length of deliberation needed?* What are the rules of deliberation? - If deliberation is used to provide an opinion (eg, advice or recommendation), who has voting rights?* - How are criteria made available to guide an exchange of viewpoints?* If deliberation is used to provide an opinion (eg, advice or recommendation),” how is the deliberation to end? (closure)*	What perspectives need to be considered? Stakeholder perspectives ○ Patient(s) □ ○ Public/citizens □ ○ Providers of care □ ○ Payers/purchasers □ ○ Producers of technology □ ○ Researchers □ ○ Policy makers □ Technical perspectives— relevant experts in: ○ Medicine □ ○ Law □ ○ Ethics □ ○ Economics □ ○ Healthcare administration □ ○ Management science □ ○ Epidemiology □ ○ Patient and/or public involvement and engagement □ ○ Bioengineering □ ○ Political science □ ○ Sociology □ ○ Anthropology □ ○ Psychology □ ○ Statistics □ ○ Other, please specify □ Standing membership of participants ○ Fixed-term membership □ ○ Renewable membership with limit □ ○ Renewable membership with no limit □ ○ Other, please specify □ Invited participants per meeting □ Mix of both □ Open to all (public call) □ Open to all who qualify (application process) □ Nominated by relevant interest groups (nomination process) □ By invitation or appointment (closed procedure) □ Using a hybrid approach □ Each participant provides their own point of view □ Each participant represents the views of others (delegates) □ Participants represent others, but are free to express individual views as they see fit (trustees) □ Mix of these □ Publicly (name and affiliation) □ Publicly (name, affiliation, and conflicts of interests) □ Not identified (anonymous) □ Open to the public □ Closed to the public □ Open only to selected individuals or groups □ Open in part and closed in part □ Face-to-face deliberation □ Virtual deliberation (eg, video and/or teleconference) □ Face to face only for selected individuals □ Written (eg, email and online forums) □ At a single meeting □ Over several meetings, with participants exchanging views at the same time □ At one or several meetings with participants exchanging views at different times (eg, Delphi process) □ All participants □ Selected participants (eg, standing □ Other, please specify □ Explicit criteria are available only to participants. □ Explicit criteria are publicly available. □ Explicit criteria are not available. □ Consensus-based procedure □ Voting procedure □ A mix of these □ Other, please specify □	Participants must be able to exchange views with each other. Although they may also share information, this may be subject to rules. (See “What information will be made available to the deliberative process?”) If a wide range of views exists within any expert or stakeholder category, care should be taken to identify an appropriate number of participants, as well as, ensure that the range of views is adequately represented. (See “How are perspectives represented?”) Identity as a stakeholder does not necessarily entitle participation. For example, the maker of a product being evaluated is certainly a stakeholder and could be allowed to exchange views, but has a direct conflict of interest in creating recommendations. Beyond membership in a deliberative process, HTA bodies must also consider broader aspects of governance, such as to whom the deliberative group is accountable. Once needed perspectives and how they will be represented are established, the degree of transparency and type of deliberation needs to be decided. Declaring who is involved and their respective interests, as well as how conflicts will be decided and settled, will enhance the perceived legitimacy of the process. How and in what manner participants are able to exchange views can affect the level of communication and trust among participants. Some deliberative processes are used to provide an opinion (eg, advice or recommendation). There are numerous approaches to arriving at a collective opinion. These involve consensus finding, voting, or a combination of both. Any approach taken to exchange viewpoints can greatly influence the outcome. Although majority-based voting is a common approach to arriving at a collective opinion, alternative procedures, may be employed to reduce divergence in viewpoints (eg, blocking), create more transparency regarding the range of viewpoints around any opinion (eg, dissenting opinions), or reduce distrust among participants. Approaches to minimize poor decision making by groups, including nominal group techniques, consensus building approaches, and expert elicitation techniques, have been developed in past decades, but these approaches are seldom used by HTA bodies.
Supporting a deliberative process	How will the exchange of view-points be facilitated during the deliberative process? What information will be made available to the deliberative process? How will information be reviewed and revised? What information will be considered by participants? How will information be presented? What specific supports for participants are available? Are comprehensive terms of reference in place?	Via one or more central agents (eg, chairs or facilitators) □ Directly with each other □ All information made available to the HTA body □ Information summarized according to with explicit methods □ Information summarized without explicit methods □ By the HTA or decision-making body □ By participants in the deliberative □ By external stakeholders and experts □ A combination of these □ Any information provided by the HTA body and participants in the deliberative process □ Only information considered allowable by HTA body or healthcare system through an explicit method □ Only information considered allowable by HTA body or healthcare system without an explicit method □ Information determined by all participants to be allowable through deliberation □ Written material □ Visual presentation (slides or video) □ Orally □ A mix of these □ Remuneration for participants time and expenses □ Training and education □ Participant-oriented tools (eg, checklists) □ A mix of these □ Terms of reference for a deliberative process should address the key features of conducting and supporting a process (indicated with * in this Checklist).	If information is interpreted for participants, it is best to use standardized guidance and provide an overview of what information is missing. HTA bodies may consider (i) an independent review and/or (ii) collect reactions and perceptions of present this as additional information for the deliberating body. To increase perceptions of legitimacy, participants should have access to all information equally. Meaningful participation requires strong engagement and a clear and common understanding of the available information and its interpretation. A checklist for HTA bodies to design their efforts for meaningful stakeholder participation has been developed (12). Training and education should be considered so that individuals can participate fully in an informed deliberation. To enhance public trust and be transparent, the terms of reference should be available and publicly accessible.
Development and communication of the output(s) of deliberation	How will the process and output (s) of a deliberation be reported and communicated? How will the outcomes of deliberation be reconsidered? How will the final output(s) of deliberation be communicated?	Recording (video, audio, or transcript) □ Written report—interpreted and summarized from the recording (eg, minutes) □ One or more of these □ Additional deliberation □ Appeal of an opinion. Characteristics of an appeal process should include: ○ What constitutes grounds for appeal and/or revision □ ○ The characteristics of the body in charge of the appeal □ ○ Who can or cannot appeal □ ○ How the appeal will be considered, decided, and communicated □ ○ The timelines involved □ A mix of these □ Broadly, not targeting specific stakeholder groups □ Narrowly, targeting specific stakeholder groups □	Consideration should be given to the timeliness, completeness, and level of complexity of information communicated. Providing a complete record can facilitate a better understanding of situation-specific considerations that may arise within deliberation and that may ultimately affect the output of the HTA process. In developing a communication strategy, be explicit about what, when, how, and to whom the output(s) will be communicated and that they will be publicly available.
Monitoring and evaluating a deliberative process	Is the desired change(s) from implementing a deliberative process established? How does the deliberative process align with the health system values or principles established and/or with those of the HTA body? How will the desired change(s) from implementing a deliberative process be measured? How will the desired change(s) from implementing a deliberative process be assessed? What indicators will be used to monitor and evaluate the deliberative process?	Yes, an explicit, comprehensive description of the desired change(s) was established prior to deliberation. □ Yes, a description of the desired change (s) was established after deliberation. □ No, a description of the desired change (s) was not established. Please specify why it was not established. □ The deliberative process aligns with the health system values or principles established and/or with those of the HTA body. □ The deliberative process partially aligns with the health system values or principles established and/or with those of the HTA body. □ Using objective measures (eg, time and resource use) □ Input from stakeholders (eg, surveys, interviews, and/or focus groups) □ Input from participants of the deliberative process after deliberation □ A hybrid of these □ Routinely and embedded in an assessment framework for the HTA body □ Routinely but only dedicated to the deliberative process □ On an ad hoc basis □ Process indicators (eg, timeliness and verifiability) □ Outcome indicators (eg, sense of ownership, opportunities to provide input, and acceptance of decisions) □ Both of these □	

**Table 2:** Monitoring and Evaluation of Deliberation in health technology assessment (HTA)

Indicator	Whom to involve	Proposed methods
Process indicators		
Transparency	Stakeholders^a^	Self-administered questionnaire, interview, focus group discussion, and/or survey among stakeholders (not) directly involved in the process
Impartiality		
Inclusivity		
Timeliness		
Consistency		
Verifiability		
Outcome indicators		
Sharing and expansion of viewpoints, (better) understanding of preferences, or the relative weight of preferences	Researchers and evidence users	Document review (minutes of meetings/video analysis), interview, or survey
Increased sense of belonging/ownership	Stakeholders (involved in the process)	Interview, survey, or direct observation
Improved capacity for deliberation	Researchers, stakeholders, and evidence users	Interview, survey, or direct observation
Increase of public trust including promotion of the legitimacy of decisions, as well as their communication	Stakeholders^a^ and general public^b^	Interview, or survey (opinion polls)
Improvement of the use of evidence including enlarging the range of relevant empirical material admissible as evidence	Researchers and evidence users	Document review, interview, or survey
Strengthening of integrity by limiting the effects of self-interest	Researchers, stakeholders (involved in the process), and evidence users	Interview or survey
Reasons provided for decisions, and the potential adjustment of decisions following deliberation	Evidence users	Policy discourse analysis
Greater acceptance of decisions	Stakeholders and general public	Survey, policy analysis, and observation on contradictory movement
Efficiency of the deliberative process considering financial resources spent against the deliberative outcomes	Funders and stakeholders (involved in the process)	Costing study, economic evaluation, interview, or survey

*Notes. Researchers* refer to those generating or synthesizing evidence for HTA. *Evidence users* are policy makers considering HTA evidence.

a *Stakeholders* include those interested in the HTA process and/or HTA-informed policy decisions with or without direct involvement in the HTA process.

b *General public* refer to members of society who have no special role in HTA, but may benefit from the outcomes of the processes.

## Box 1 HTA Processes Where Deliberation May Take Place

1. Governance and structure of an HTA program (deliberation about processes)
2. At HTA-related stages (deliberation within processes)
  - Identification of topics (eg, horizon scanning)
  - Prioritization of relevant topics for HTA
  - Providing scientific advice
  - Scoping, assessment, and synthesis of relevant information
  - Contextualization of HTA
  - Development and communication of the output(s)
  - Monitoring and evaluation

## Box 2 Definitions

Deliberation in HTA is the informed and critical examination of an issue and the weighing of arguments and evidence to guide a subsequent decision.

A deliberative process for HTA consists of procedures, activities, and events that support deliberation in HTA.

## Box 3 Examples of Deliberation at Different HTA Stages

### Identification and prioritization of relevant topics for HTA

In Thailand, twice a year, the HTA body invites representatives of relevant stakeholders, such as policy makers, health professionals, academics, patient associations, industry, civil society, and the public to suggest topics for assessment. This process involves a multistage process of deliberation (in the form of a working group comprised of stakeholders who have their own stakeholder group deliberations prior to nominating the topics to the working group) informed by evidence and the use of explicit criteria, such as size of the affected population, severity of disease, effectiveness of interventions, variation in practice, economic impact on household expenditure, and ethical and social implications.^21,22^

### Providing scientific advice to technology developers

The National Institute for Health and Care Excellence (NICE) in England provides scientific advice to developers of drugs, medical devices, biotechnology, digital health technologies, and diagnostics regarding their evidence-generating plans needed for HTA. A formal process is in place in which NICE employees work together with independent clinical and health economic experts, as well as carers and/or patient experts. The participation of patients and/or carers provides an opportunity to understand what is important to patients about their condition and their treatment. All experts equally participate in developing the advice, and support mechanisms are available. Due to the confidential nature of the service, the meeting with the technology developer is not open to the public, not recorded, filmed, or minuted. After the meeting, experts are asked to confirm statements attributed to them. For more information: https://www.nice.org.uk/about/what-we-do/life-sdences/scientific-advice.

### Scoping, assessment, and synthesis of information

Brereton et al^23^ explored the role of different stakeholders and experts in scoping an HTA on palliative home care in seven European countries (England, Germany, Italy, Lithuania, The Netherlands, Norway, and Poland). They included 132 stakeholders and experts, of which 80 were commissioners, health and social care professionals/academics working in palliative care, and 52 comprised lay persons, including patients and families undergoing palliative care of 18 years and older. The contributions enabled the researchers to identify both an intervention and comparator model of service provision for the main HTA question, as well as to inform subquestions for the assessment of specific aspects (eg, ethical and sociocultural aspects).The Mathematical and Economic Modelling for Vaccination and Immunisation Evaluation Program in the United Kingdom is the first initiative to have the public participate in mathematical and economic modeling. For this purpose, a public involvement reference group deliberated with the academic researchers about different forms of knowledge, expertise, and evidence. The program indicated that by using deliberation, assumptions of the model could be challenged, and its validity enhanced.^24^

### Contextualization of HTA

In 2017, the Ministry of Health and Social Services in Quebec (Canada) requested the provincial HTA institute (Institut National d’Excellence en Santé et Service Sociaux [INESSS]) to develop recommendations regarding the diagnosis, treatment, and follow-up of Lyme disease. The INESSS evaluated different modalities of patient engagement, such as consultation, as well as participation (ie, 2 patients participated in all phases of the HTA). They found that patients on the advisory committee participated in the discussions and were able to influence decisions by sharing their experiences.^25^

## Box 4 Consideration of Stakeholder Influence

During the 2017 Health Technology Assessment International Latin America Policy Forum, more than 40 representatives of HTA bodies, governments, social security and private insurance sectors, industry, academics, and NGOs discussed stakeholder involvement in HTA processes. Participants expressed concern that certain groups (eg, industry) could have an excessive influence on setting the agenda for prioritization of technologies to be assessed. They also mentioned that stakeholders could inappropriately influence the assessment and decision making as well, and presented cases of pressures received from severely ill patients or patient groups highly associated with industry. This concern was more pronounced in countries with a lower level of HTA institutionalization where HTA mechanisms and structures are weaker or in the process of emerging.^28^

## Box 5 Understanding the Role of Contextual Factors Specific to a Health Technology

Kleinhout-Vliek et al^32^ studied the use of contextual factors in health coverage decision making for four health technologies (nivolumab, benzodiazepines, smoking cessation, and walking aid with wheels) in Belgium, England, Germany, and The Netherlands. Contextual factors were operationalized as arguments about necessity (eg, disease severity, unmet medical need, dignity, and human rights) because their perceived validity varies per health technology and because they are elements not easily quantified in clinical and/or economic assessment. These factors are actively used in deliberations, in addition to decision criteria (eg, effectiveness and cost effectiveness). In some countries, they are part of the set of formalized criteria (individual burden of disease in England, Germany, and The Netherlands; and individual cost considerations in The Netherlands). The number of contextual factors considered varied per country and per case: from 6 different factors (Germany) to sixteen factors (The Netherlands). Some contextual factors were used implicitly, in the deliberation and/or related documents. Others were made explicit and stated in decision documents. In addition, half of the decisions were taken in other settings than the HTA body, with documents that should provide a justification for the final decision being absent. The latter is important because of transparency and accountability reasons.

## Box 7 Example of Committee Composition in Ontario

An example of different committee composition within the same political context based on differing mandates is seen in the province of Ontario (Canada). The Ontario Health Ministry hosts two separate HTA processes for drug and nondrug decision making. Drug funding recommendations in 2018 involved deliberation across sixteen voting experts, representing practicing pharmacists, medical specialists, and health economists, along with an expert in patient experience who participated in regular meetings. There was no broader stakeholder consultation to inform the deliberations. The nondrug deliberation occurred across a group of up to twenty voting members having similar expertise as the drug committee, although this committee also includes experts in ethics, and a minimum of two members contributed to patient and public perspectives, and one of them had current or recent experience in industry.

## Box 8 National Institute for Health and Care Excellence’s (NICE) Appeal Process

An appeal can be lodged by any of the appraisal consultees and can be considered at an oral hearing or by written submission. Consultees have 15 working days to make an appeal, starting from the day the final draft guidance is issued to consultees and commentators. Appeals must be submitted in writing and must fall within one or more of the 2 grounds of appeal. The grounds of appeal are:

(1) In making the assessment that preceded the recommendation, the NICE has (i) failed to act fairly or (ii) exceeded its powers.
(2) The recommendation is unreasonable in the light of the evidence submitted to NICE.

The Vice Chair of the NICE reviews lodged appeals and considers whether they fall within the grounds for appeal. If they do, and are “arguable,” the Vice Chair will decide if an oral or written appeal hearing will be held. The aim is to hold hearings within 8 weeks of the end of the appeal period for oral hearing and 10 weeks for written submissions.

A panel is convened to hear the appeal. The panel is drawn from a group of people approved by the Secretary of State for Health and Social Care to hear appeals. Each appeal panel consists of five members, four of whom are independent of the NICE. An external member will chairthe appeal panel. This chair will be either: engaged in the provision of healthcare in the NHS, someone with experience in representing patients or carers, or a patient or carer (patient representative). The panel will include an NHS representative, a representative of the life sciences industry, a patient representative, and a nonexecutive director of the NICE.

An overview of health technologies for which an appeal was issued, as well as an overview of the complete appeal process, can be found on the NICE Web site: www.nice.org.uk/about/what-we-do/our-programmes/nice-guidance/nice-technology-appraisal-guidance/technology-appraisal-and-highly-specialised-technologies-appeals (source of this Box text)

## Box 9 Key Reporting Items to Communicate a Recommendation or Decision as the Final Output of Deliberation

- The context of the decision (ie, any legislative or regulatory underpinning of the role of the committee making the decision or recommendation);
- What the decision was and what options or alternatives were considered;
- What facts were used and what were the reasons for taking into account certain information, but also the reasons for excluding information;
- Who was involved in making the decision as a participant in the process;
- Who has written the recommendation and/or decision;
- Why the decision as described was made (ie, underlying rationale);
- If and how the decision will feed into the policy process and relate to decision making, either on national, regional, or local level?

## References

[1] Kolotourou K, Ermacora P, Grosvenor A (2019). The evolution of European HTA and access to innovative medicines. J Comp Eff Res.

[2] Abelson J, Forest PG, Eyles J (2003). Deliberations about deliberative methods: Issues in the design and evaluation of public participation processes. Soc Sci Med.

[3] Lomas J, Culyer AJ, McCutcheon C, McAuley L, Tetroe J (2005). Conceptualizing and combining evidence for health system guidance. Final report.

[4] Dobrow MJ, Chafe R, Burchett HED, Culyer AJ (2012). Designing deliberative methods for combining heterogeneous evidence: A systematic review and qualitative scan. Research conducted: 2009.

[5] Bond K, Stiffell R, Ollendorf D (2020). Principles for deliberative processes in health technology assessment. Int J Technol Assess Health Care.

[6] Oortwijn W, Jansen M, Baltussen R (2021). A practical guide for HTA bodies for legitimate benefit package design.

[7] Kristensen FB, Husereau D, Huic M (2019). Identifying the need for good practices in health technology assessment: Summary of the ISPOR HTA council working group report on good practices in HTA. Value Health.

[8] Malone DC, Ramsey SD, Patrick DL (2020). Criteria and process for initiating and developing an ISPOR good practices task force report. Value Health.

[9] Gauvin FP (2009). What is a deliberative process? Factsheet.

[10] Fearon JD, Elster J (1998). Deliberative democracy.

[11] Hemmati M (2007). Participatory dialogue: Towards a stable, safe and just society for all.

[12] Jansen MPM, Baltussen R, Bærøe K (2018). Stakeholder participation for legitimate priority setting: A checklist. Int J Health Policy Manag.

[13] Boothe K (2019). “Getting to the table”: Changing ideas about public and patient involvement in Canadian drug assessment. J Health Polit Policy Law.

[14] (2020). Innovative citizen participation and new democratic institutions: Catching the deliberative wave.

[15] Solomon S, Abelson J (2012). Why and when should we use public deliberation?. Hastings Cent Rep.

[16] Yesilkagit K, Christensen JG (2010). Institutional design and formal autonomy: Political versus historical and cultural explanations. J Public Adm Res Theory.

[17] Landwehr C, Böhm K (2011). Delegation and institutional design in healthcare rationing. Governance.

[18] Abelson J, Wagner F, DeJean D (2016). Public and patient involvement in health technology assessment: A framework for action. Int J Technol Assess Health Care.

[19] Glassman A, Chalkidou K, Giedion U (2012). Priority-setting institutions in health: Recommendations from a center for global development working group. Glob Heart.

[20] Culyer AJ (2009). Deliberative processes in decisions about health care technologies: Combining different types of evidence, values, algorithms and people.

[21] Leelahavarong P, Doungthipsirikul S, Kumluang S (2019). Health technology assessment in Thailand: Institutionalization and contribution to healthcare decision making: Review of literature. Int J Technol Assess Health Care.

[22] Tantivess S, Doungthipsirikul S (2019). Leaving no one behind: Participatory technology appraisal as a platform for agenda setting to address disparities in access to health services in Thailand. Int J Technol Assess Health Care.

[23] Brereton L, Ingleton C, Gardiner C (2017). Lay and professional stakeholder involvement in scoping palliative care issues: Methods used in seven European countries. Palliat Med.

[24] Staniszewska S, Hill EM, Grant R (2021). Developing a framework for public involvement in mathematical and economic modelling: Bringing new dynamism to vaccination policy recommendations. Patient.

[25] Pomey MP, Bush PL, Demers-Payette O (2021). Developing recommendations for the diagnosis and treatment of Lyme disease: The role of the patient’s perspective in a controversial environment. Int J Technol Assess Health Care.

[26] Pichon-Riviere A, Augustovski F, García Martí S, Alfie V, Sampietro-Colom L (2020). The link between health technology assessment and decision making for the allocation of health resources in Latin America. Int J Technol Assess Health Care.

[27] Kapiriri L, Baltussen R, Oortwijn W (2020). Implementing evidence-informed deliberative processes in health technology assessment: A low income country perspective. Int J Technol Assess Health Care.

[28] Pichon-Riviere A, Soto N, Augustovski F, Sampietro-Colom L (2018). Stakeholder involvement in the health technology assessment process in Latin America. Int J Technol Assess Health Care.

[29] Mohara A, Youngkong S, Velasco RP (2012). Using health technology assessment for informing coverage decisions in Thailand. J Comp Eff Res.

[30] Vanstone M, Abelson J, Bidonde J (2019). Ethical challenges related to patient involvement in health technology assessment. Int J Technol Assess Health Care.

[31] Pichon-Riviere A, Soto NC, Augustovski FA, Garcia Marti S, Sampietro-Colom L (2018). Health technology assessment for decision making in Latin America: Good practice principles. Int J Technol Assess Health Care.

[32] Kleinhout-Vliek T, de Bont A, Boysen M (2020). Around the tables–Contextual factors in healthcare coverage decisions across Western Europe. Int J Health Policy Manag.

[33] Ten Have A, Oortwijn W, Broos P, Nelissen E European cooperation on health technology assessment. Economic and governance analysis of the establishment of a permanent secretariat. Final report.

[34] Boers M (2020). Governance of European cooperation processes in health technology assessment. Networking, paving the way to convergence of practices? Thesis.

[35] Clark S, Weale A (2012). Social values in health priority setting: A conceptual framework. J Health Organ Manag.

[36] Walton MJ, O’Connor J, Carroll C, Claxton L, Hodgson R (2019). A review of issues affecting the efficiency of decision making in the NICE single technology appraisal process. Pharmacoecon Open.

[37] European Network for Health Technology Assessment (2017). An analysis of HTA and reimbursement procedures in EUnetHTA partner countries: Final report.

[38] Cesnulaityte I (2020). Innovative citizen participation and new democratic institutions: Catching the deliberative wave.

[39] Bijlmakers L, Jansen M, Boer B (2020). Increasing the legitimacy of tough choices in healthcare reimbursement: Approach and results of a citizen forum in the Netherlands. Value Health.

[40] Moynihan R, Oxman AD, Lavis J, Paulsen E (2006). A review of organizations that support the use of research evidence in developing guidelines, technology assessments, and health policy, for the WHO Advisory Committee on Health Research.

[41] Rothwell JD (2013). mixed company: Communicating in small groups and teams.

[42] Wranik WD, Zielinska DA, Gambold L, Sevgur S (2019). Threats to the value of health technology assessment: Qualitative evidence from Canada and Poland. Health Policy.

[43] Fox J, Shotts KW (2009). Delegates or trustees? A theory of political accountability. J Polit.

[44] Wortley S, Tong A, Howard K (2017). Community views and perspectives on public engagement in health technology assessment decision making. Aust Health Review.

[45] Werkö SS, Staniszewska S (2021). Patient and public involvement in health technology assessment: A new dawn?. Int J Technol Assess Health Care.

[46] Bidonde J, Vanstone M, Schwartz L, Abelson J (2021). An institutional ethnographic analysis of public and patient engagement activities at a national health technology assessment agency. Int J Technol Assess Health Care.

[47] Bombard Y, Abelson J, Simeonov D, Gauvin FP (2011). Eliciting ethical and social values in health technology assessment: A participatory approach. Soc Sci Med.

[48] Boivin A, Lehoux P, Burgers J, Grol R (2014). What are the key ingredients for effective public involvement in health care improvement and policy decisions? A randomized trial process evaluation. Milbank Q.

[49] Chwalisz C (2020). Innovative citizen participation and new democratic institutions: Catching the deliberative wave.

[50] Mendonça RF, Ercan SA, Asenbaum H (2022). More than words: A multidimensional approach to deliberative democracy. Polit Stud.

[51] Peel A, Jenks M, Choudhury M (2018). Use of expert judgement across NICE guidance-making programmes: A review of current processes and suitability of existing tools to support the use of expert elicitation. Appl Health Econ Health Policy.

[52] Janis IL (1982). Groupthink: Psychological studies of policy decisions and fiascoes.

[53] Gibson JL, Martin DK, Singer PA (2005). Priority setting in hospitals: Fairness, inclusiveness, and the problem of institutional power differences. Soc Sci Med.

[54] Schlander M (2021). HTA agencies need evidence-informed deliberative processes: Comment on “Use of evidence-informed deliberative processes by health technology assessment agencies around the globe”. Int J Health Policy Manag.

[55] Shayo EH, Norheim OF, Mboera LEG (2012). Challenges to fair decision-making processes in the context of health care services: A qualitative assessment from Tanzania. Int J for Equity Health.

[56] Kao AB, Berdahl AM, Hartnett AT (2018). Counteracting estimation bias and social influence to improve the wisdom of crowds. J R Soc Interface.

[57] Kahan JP, Morton SC, Farris HH, Kominski GF, Donovan AJ (1994). Panel processes for revising relative values of physician work. A pilot study Med Care.

[58] Simera I, Moher D, Hoey J, Schulz KF, Altman DG (2009). The EQUATOR Network and reporting guidelines: Helping to achieve high standards in reporting health research studies. Maturitas.

[59] Wale JL, Thomas S, Hamerlijnck D, Hollander R (2021). Patients and public are important stakeholders in health technology assessment but the level of involvement is low-A call to action. Res Involv Engagem.

[60] International Association for Public Participation (2015). Australasia. Quality Assurance Standard in Community and Stakeholder Engagement.

[61] Bae EY, Hong JM, Kwon HY (2016). Eight-year experience of using HTA in drug reimbursement: South Korea. Health Policy.

[62] Sibbald SL, Singer PA, Upshur R, Martin DK (2009). Priority setting: What constitutes success? A conceptual framework for successful priority setting. BMC Health Serv Res.

[63] Mercer RE, Chambers A, Mai H (2020). Are we making a difference? A qualitative study of patient engagement at the pan-Canadian oncology drug review: Perspectives of patient groups. Value Health.

[64] Oortwijn W, Determann D, Schiffers K, Tan SS, van der Tuin J (2017). Towards integrated health technology assessment for improving decision making in selected countries. Value Health.

